# Primary bacterial intercostal pyomyositis diagnosis: A case report

**DOI:** 10.1097/MD.0000000000033723

**Published:** 2023-05-05

**Authors:** Hirokazu Toyoshima, Motoaki Tanigawa, Chiaki Ishiguro, Hiroyuki Tanaka, Yuki Nakanishi, Shigetoshi Sakabe, Junzo Hisatsune, Shoko Kutsuno, Yasuhisa Iwao, Motoyuki Sugai

**Affiliations:** a Department of Infectious Diseases, Japanese Red Cross Ise Hospital, Ise, Japan; b Department of Respiratory Medicine, Japanese Red Cross Ise Hospital, Ise, Japan; c Department of Medical Technology, Japanese Red Cross Ise Hospital, Ise, Japan; d Antimicrobial Resistance Research Center, National Institute of Infectious Diseases, Higashimurayama, Japan.

**Keywords:** blood culture, case report, intercostal muscle, magnetic resonance imaging, primary pyomyositis, single-nucleotide polymorphism analysis, *Staphylococcus aureus*, ultrasonography, whole-genome sequencing

## Abstract

Pyomyositis is a microbial infection of the muscles and contributes to local abscess formation. *Staphylococcus aureus* frequently causes pyomyositis; however, transient bacteremia hinders positive blood cultures and needle aspiration does not yield pus, especially at the early disease stage. Therefore, identifying the pathogen is challenging, even if bacterial pyomyositis is suspected. Herein, we report a case of primary pyomyositis in an immunocompetent individual, with the identification of *S aureus* by repeated blood cultures.

**Patient concerns:**

A 21-year-old healthy man presented with fever and pain from the left chest to the shoulder during motion. Physical examination revealed tenderness in the left chest wall that was focused on the subclavicular area. Ultrasonography showed soft tissue thickening around the intercostal muscles, and magnetic resonance imaging with short-tau inversion recovery showed hyperintensity at the same site. Oral nonsteroidal anti-inflammatory drugs for suspected virus-induced epidemic myalgia did not improve the patient’s symptoms. Repeated blood cultures on days 0 and 8 were sterile. In contrast, inflammation of the soft tissue around the intercostal muscle was extended on ultrasonography.

**Diagnoses:**

The blood culture on day 15 was positive, revealing methicillin-susceptible *S aureus* JARB-OU2579 isolates, and the patient was treated with intravenous cefazolin.

**Interventions:**

Computed tomography-guided needle aspiration from the soft tissue around the intercostal muscle without abscess formation was performed on day 17, and the culture revealed the same clone of *S aureus*.

**Outcomes:**

The patient was diagnosed with *S aureus*-induced primary intercostal pyomyositis and was successfully treated with intravenous cefazolin for 2 weeks followed by oral cephalexin for 6 weeks.

**Lessons:**

The pyomyositis-causing pathogen can be identified by repeated blood cultures even when pyomyositis is non-purulent but suspected based on physical examination, ultrasonography, and magnetic resonance imaging findings.

## 1. Introduction

Pyomyositis is an acute or subacute bacterial infection of the skeletal muscles that is commonly accompanied by abscess formation.^[1,2]^ This infection has been classically reported in tropical areas such as sub-Saharan Africa; however, it has been increasingly reported even in temperate regions.^[1]^ *Staphylococcus aureus* is the most common pathogen that causes pyomyositis. Predisposing risk factors for pyomyositis include immunodeficiency virus infection, diabetes mellitus, alcoholic liver disease, corticosteroid therapy, hematologic malignancies, renal failure, autoimmune diseases, trauma, and rigorous exercise.^[1]^ Blood culture is positive only in 5% to 35% of cases; therefore, identifying the pathogen is often challenging in cases without abscess formation.^[3]^ Herein, we report a case of primary pyomyositis in an immunocompetent individual, with identification of *S aureus* by repeated blood cultures.

## 2. Case presentation

A 21-year-old previously healthy and non-obese man, who worked as a deliveryman, presented with a low-grade fever and pain from the left chest to the shoulder during motion for 7 days.

**Figure 1.**
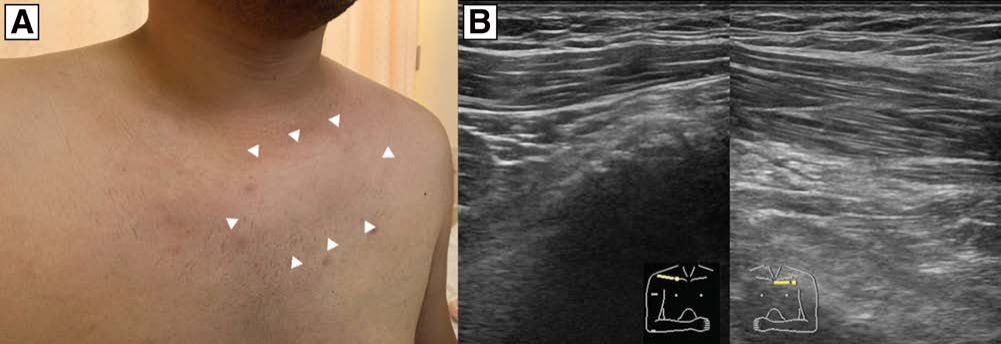
Physical examination and ultrasound findings. (A) Physical examination showed swelling of the left upper chest wall (white arrowheads) with pain triggered by left shoulder movement or deep breathing. (B) Ultrasound study revealed swollen upper intercostal soft tissue under the left pectoralis major muscle.

**Figure 2.**
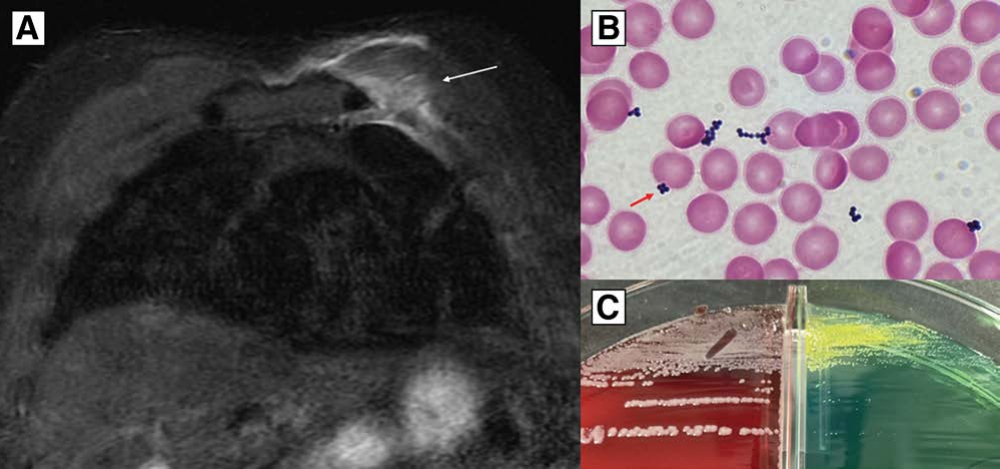
MRI and microbiological findings. (A) Coronal MRI with short-tau inversion recovery showed high intensity (white arrow) at the same part, consistent with the finding in ultrasonography. (B) Repeated blood cultures drawn on days 0 and 8 were negative; however, blood culture on day 15 (22 d after the appearance of symptoms) revealed positive for clusters of gram-positive cocci (red arrow, Gram staining, ×1000). (C) Colonies identified as methicillin-susceptible *Staphylococcus aureus* grew on 5% sheep blood agar and bromothymol blue lactose agar. MRI = magnetic resonance imaging.

His vital signs were as follows: temperature, 36.6°C; blood pressure, 140/76 mm Hg; heart rate, 80 beats/min; respiratory rate, 15 breaths/min; and oxygen saturation, 96% in ambient air. Physical examination revealed slight swelling in the left chest wall and tenderness in the left chest wall that was focused on the subclavicular area (Fig. 1A). The laboratory findings were as follows: C-reactive protein, 2.20 mg/dL; erythrocyte sedimentation rate, 13 mm/h; creatine kinase, 72 U/L; lactate dehydrogenase, 150 U/L; and white blood cell count, 7100/µL with 62.6% neutrophils. Administration of oral nonsteroidal anti-inflammatory drugs for 9 days for the suspected virus-induced epidemic myalgia did not improve the patient’s symptoms. Ultrasonography revealed soft tissue thickening around the intercostal muscles (Fig. 1B). In addition, magnetic resonance imaging (MRI) with short-tau inversion recovery revealed an area with hyperintensity (Fig. 2A). Repeated blood cultures on days 0 and 8 were sterile; however, inflammation of the soft tissue around the intercostal muscle was extended on ultrasonography. The blood culture on day 15 (22 days after the appearance of symptoms) was positive for gram-positive cocci (Fig. 2B and C) and revealed *mecA*-negative *S aureus* JARB-OU2579 using Verigene^®^ (Luminex, Austin, TX), which is a rapid microbial detection system. The patient was treated with intravenous cefazolin (2 g) every 8 hours. Computed tomography (CT)-guided needle aspiration from the soft tissue around the intercostal muscle without abscess formation was performed on day 17, and the culture revealed *S aureus* JARB-OU2580. We performed whole-genome sequencing to analyze the genotyping of these strains. Genomic DNA was purified from the culture using lysostaphin and the AMPure XP system (Beckman Coulter Inc., Brea, CA), according to the manufacturer’s instructions. Sequencing libraries were prepared as previously described,^[4]^ and paired-end sequencing (2 × 300 bp) was performed on the Illumina MiSeq platform (Illumina Inc., San Diego, CA). The sequence data have been deposited in the DDBJ Sequence Read Archive under accession number DRR441031–DRR441034. Furthermore, *S aureus* JARB-OU2579 isolated from blood culture and JARB-OU2580 isolated from needle aspiration culture were ST8, *mecA*-negative, Panton-Valentine leukocidin (PVL) gene-negative, *blaZ*-positive, *sep*-positive, and *sel*-positive. Additionally, single-nucleotide polymorphism analysis suggested that the number of single-nucleotide polymorphisms between JARB-OU2579 and JARB-OU2580 was only three, indicating that they were the same clone.

The patient was diagnosed as having primary intercostal pyomyositis caused by *S aureus*, and he was successfully treated with intravenous cefazolin (2 g) every 8 hours for 2 weeks followed by oral cephalexin (2000 mg) every day for 6 weeks. The follow-up blood culture was negative on day 18. There was no evidence of infective endocarditis on transthoracic echocardiography or disseminated foci on contrast-enhanced whole-body CT. The swelling and pain in the left chest wall improved gradually. To date, the patient remains disease free without recurrence.

## 3. Discussion and conclusions

This case illustrates 4 clinical issues as follows: the possibility of primary pyomyositis of the chest wall in immunocompetent young adults in the absence of any predisposing factors, the clinical benefit of ultrasound imaging as a point-of-care test, optimal methods to identify the pathogens in cases with primary pyomyositis, and clinical management of primary pyomyositis caused by *S aureus*.

Pyomyositis frequently occurs in large muscles of the lower extremities. In contrast, chest pyomyositis accounts for only 6% of all pyomyositis cases.^[5]^ The patient had intercostal pyomyositis, which is a rare site of pyomyositis, without the presence of any predisposing factors. A history of preceding muscle injury, such as trauma, has been reported in pyomyositis.^[6]^ In contrast, Maravelas et al^[7]^ reported that most cases of pyomyositis occur without an adjacent source of infection. The patient had no history of trauma, and physical examination revealed no evidence of an apparent wound. Therefore, the patient may have experienced unrecognized trauma to the left intercostal muscle, since he worked as a delivery man. These findings suggest that primary pyomyositis can occur even in healthy young adults without any predisposing factors.

MRI is the gold standard for diagnosing pyomyositis.^[8]^ However, ultrasound imaging has been recognized as an appropriate tool, since it reveals muscle enlargement and changes in echogenicity due to local inflammation or abscess formation.^[8]^ Unlike MRI, ultrasound imaging does not require expensive equipment, but it provides helpful information for the diagnosis of pyomyositis. In our case, pyomyositis was suspected based on ultrasound imaging and physical examination.

The most common pathogen causing pyomyositis is *S aureus* (66%); however, *Streptococcus* species (11%), *Streptococcus pyogenes* (4.8%), *S agalactiae* (3.6%), *Pseudomonas* species (3.6%), *Enterococcus* species (2.7%), and *Escherichia coli* (2.7%) can also cause pyomyositis.^[7]^ Therefore, identification of the pathogen contributes to improvement in the prognosis of patients with pyomyositis. Nevertheless, blood culture is positive only in 5% to 35% of pyomyositis cases because of transient bacteremia in pyomyositis.^[3]^ In addition, needle aspiration does not yield pus in the early phase.^[6,9]^ The clinical course of pyomyositis is divided into 3 stages.^[3,6,9]^ The first stage (invasive stage) is characterized by cramping pain that is followed by edema and low-grade fever with mild leukocytosis 1 week later. The second stage (suppurative stage) occurs after 1 to 3 weeks of the symptoms in the first stage, and it is characterized by noticeable edema, muscular tenderness, and a middle-grade fever with leukocytosis. Needle aspiration can yield pus during this stage. The third stage (late stage) is a severe condition that is characterized by sepsis, fluctuating tenderness, and high-grade fever. In our case, blood cultures taken twice in the first stage were negative; however, repeated blood cultures taken at the end of the first stage (22 days after the appearance of symptoms) were positive for *S aureus*. CT-guided needle aspiration culture 2 days after a positive blood culture also revealed the presence of the same clone of *S aureus*. Repeated blood cultures should be considered a minimally invasive tool to identify pyomyositis pathogens. Nevertheless, needle aspiration should be considered in cases with repeated negative blood cultures.

Although the optimal duration of antimicrobial treatment remains unclear, Shittu et al^[8]^ reported that antimicrobials should be administered for several weeks (2–9 weeks). Additionally, protein synthesis inhibitors, including clindamycin, can inhibit bacterial toxin production because PVL plays an important role in pyomyositis.^[8]^ In our case, the strain was PVL-negative *S aureus*; therefore, the patient was treated with 2 weeks of intravenous cefazolin, followed by 6 weeks of oral cephalexin without adjunctive clindamycin. It has been reported that after the second stage (suppurative stage), drainage of the intramuscular abscess is required in addition to appropriate antimicrobial administration.^[10]^ Herein, the patient was accurately diagnosed with repeated blood cultures and was successfully treated without drainage.

A limitation of this case report is that the precise time when blood cultures became positive is unknown since they were not obtained from days 9 to 14. Thus, it could have been possible to identify the pathogen earlier.

In conclusion, repeated blood cultures can help identify the pathogen even in the first stage of pyomyositis. This would contribute to appropriate antimicrobial treatment and prevent progression to the late stage of pyomyositis.

## Acknowledgments

We would like to thank Editage [http://www.editage.com] for editing and reviewing this manuscript for English language.

## Author contributions

**Conceptualization:** Hirokazu Toyoshima, Yuki Nakanishi, Junzo Hisatsune.

**Data curation:** Hirokazu Toyoshima, Chiaki Ishiguro, Yuki Nakanishi, Junzo Hisatsune, Shoko Kutsuno, Yasuhisa Iwao, Motoyuki Sugai.

**Funding acquisition:** Motoyuki Sugai.

**Methodology:** Hirokazu Toyoshima, Hiroyuki Tanaka, Junzo Hisatsune, Shoko Kutsuno, Yasuhisa Iwao, Motoyuki Sugai.

**Supervision:** Motoaki Tanigawa, Shigetoshi Sakabe, Junzo Hisatsune.

**Visualization:** Hirokazu Toyoshima.

**Writing – original draft:** Hirokazu Toyoshima.

**Writing – review & editing:** Hirokazu Toyoshima, Junzo Hisatsune.
